# *ARID1A* Mutations and PI3K/AKT Pathway Alterations in Endometriosis and Endometriosis-Associated Ovarian Carcinomas

**DOI:** 10.3390/ijms140918824

**Published:** 2013-09-12

**Authors:** Eleftherios P. Samartzis, Aurelia Noske, Konstantin J. Dedes, Daniel Fink, Patrick Imesch

**Affiliations:** 1 Division of Gynecology, University Hospital Zurich, Frauenklinikstrasse 10, Zurich CH-8091, Switzerland; E-Mails: eleftherios.samartzis@usz.ch (E.P.S.); konstantin.dedes@usz.ch (K.J.D.); daniel.fink@usz.ch (D.F.); 2 Institute of Surgical Pathology, University Hospital Zurich, Schmelzbergstrasse 12, Zurich CH-8091, Switzerland; E-Mail: aurelia.noske@usz.ch

**Keywords:** endometriosis, ovarian clear cell carcinoma (OCCC), endometrioid ovarian carcinoma (EnOC), ARID1A, PI3K/AKT pathway, PIK3CA

## Abstract

Endometriosis is a common gynecological disease affecting 6%–10% of women of reproductive age and is characterized by the presence of endometrial-like tissue in localizations outside of the uterine cavity as, e.g., endometriotic ovarian cysts. Mainly, two epithelial ovarian carcinoma subtypes, the ovarian clear cell carcinomas (OCCC) and the endometrioid ovarian carcinomas (EnOC), have been molecularly and epidemiologically linked to endometriosis. Mutations in the gene encoding the AT-rich interacting domain containing protein 1A (*ARID1A*) have been found to occur in high frequency in OCCC and EnOC. The majority of these mutations lead to a loss of expression of the ARID1A protein, which is a subunit of the SWI/SNF chromatin remodeling complex and considered as a bona fide tumor suppressor. *ARID1A* mutations frequently co-occur with mutations, leading to an activation of the phosphatidylinositol 3-kinase (PI3K)/AKT pathway, such as mutations in *PIK3CA* encoding the catalytic subunit, p110α, of PI3K. In combination with recent functional observations, these findings strongly suggest cooperating mechanisms between the two pathways. The occurrence of *ARID1A* mutations and alterations in the PI3K/AKT pathway in endometriosis and endometriosis-associated ovarian carcinomas, as well as the possible functional and clinical implications are discussed in this review.

## 1. Introduction

The identification of recurrent somatic mutations in endometriosis-associated ovarian cancer, in particular, AT-rich interacting domain containing protein 1A (*ARID1A*) mutations [1,2], provided the first molecular evidence of a direct pathogenic link between endometriosis and certain subtypes of ovarian carcinomas [3,4].

Endometriosis is a common gynecological inflammatory disease that affects at least 6%–10% of women of reproductive age and is characterized by the presence of endometrial-like tissue outside of the uterine cavity [5,6]. The prevalence of this disease is higher in women with abdominal pain, infertility or both, where it rises to an incidence rate of 35%–50% [7]. The ectopic endometrial-like tissue is most often found in the pelvic peritoneum, the ovaries and/or the rectovaginal septum, but can also involve uncommon localizations, such as the diaphragm, pleura, pericardium or, even, brain [7,8]. The exact pathogenesis of endometriosis has as yet not been fully elucidated. One of the most widely accepted theories is the retrograde menstruation of fragments of menstrual endometrium through the fallopian tubes that may explain the more frequent cases of peritoneal and ovarian endometriosis [5]. The typical clinical symptoms of the disease consist of dysmenorrhea, dyspareunia and infertility. Current treatment options are the surgical removal of endometriotic implants and hormonal suppressant drugs [8]. The overall risk of ovarian cancer associated with endometriosis can be regarded as generally very low [9], but is still increased compared to women not presenting with endometriosis [8].

Clinical suggestion for a causal relationship between endometriosis and ovarian cancer is not novel, and the possible pathogenic link had already been described at the beginning of the 20^th^ century by Sampson [10]. However, larger epidemiological studies showing evidence for a causal relationship between endometriosis and ovarian cancer were lacking until the end of the 20^th^ century [11].

In a large registry study among 20,686 women in Sweden who had been hospitalized for endometriosis, a significantly increased risk for ovarian cancer (standardized incidence ratio (SIR) 1.9, 95% confidence interval (CI) 1.3–2.8) was found after a mean of 11.4 years of follow-up. The risk of ovarian cancer was increased 2.5-fold in women with a follow-up of more than 10 years [12]. A larger Swedish register study involving 64,492 women with endometriosis confirmed an elevated risk of ovarian cancer in these patients with an SIR of 1.43 (95% CI 1.19–1.71). The risk of ovarian cancer was considerably higher in patients with early diagnosed and long-standing endometriosis (SIR 2.01 and 2.23, respectively) [13]. Several other studies have also described an increased risk of ovarian cancer in women with endometriosis [14–24]. A large epidemiological pooled analysis of 13 case-control studies, including 13,226 controls and 7911 women with invasive ovarian cancer, investigated the frequency of self-reported endometriosis and found significantly increased risks for ovarian cancer in women with history of endometriosis with an odds ratio of 1.46 (95% CI 1.31–1.63, *p* < 0.0001) after stratifying for age, ethnic origin and adjustment for the duration of oral contraceptive use and parity. Importantly, a significant association of the histological subtypes of clear-cell (OR 3.05, 95% CI 2.43–3.84, *p* < 0.0001), endometrioid (OR 2.04, 95% CI 1.67–2.48, *p* < 0.0001), and low-grade serous ovarian carcinomas (OR 2.11, 95% CI 1.39–3.20, *p* < 0.0001) was found with endometriosis in this study. In contrast, there was no association of endometriosis with mucinous and high-grade serous cancer, as well as borderline tumors [25].

Ovarian cancer is the most lethal gynecological neoplasm and a very heterogeneous disease [26]. Based on extensive morphologic, immunohistochemical and molecular genetic analyses of different epithelial ovarian cancer subtypes, Kurman and Shih proposed that epithelial ovarian carcinomas can be divided in two groups: Type I ovarian carcinomas comprise clear cell carcinomas (OCCC), low-grade endometrioid carcinomas (EnOC), mucinous carcinomas and low-grade serous carcinomas. They tend to be rather slow-growing, low-grade neoplasms and often develop in a stepwise manner from visible precursor lesions. On a molecular level, they often share mutations in different genes, such as *KRAS*, *BRAF*, *PTEN*, *PIK3CA*, *ARID1A*, *ERBB2*, *CTNNB1* and *PPP2R1A*. Type II ovarian carcinomas mainly cover high-grade serous carcinomas and are more frequent, as they represent approximately 75% of all ovarian carcinomas. These tumors are characterized by aggressive behavior, nearly ubiquitous presence of *TP53* mutations and a high level of genetic instability, in contrast to type I tumors, which rarely show *TP53* mutations [27–31].

There is growing evidence that epithelial ovarian carcinomas, unlike ovarian germline tumors, may often find their origin in non-ovarian tissue [32,33]. This is a shift of paradigms, since until recently, the largely accepted theory was that ovarian epithelial tumors arise from the single cell layer lining the ovarian surface, usually referred to as surface epithelium [34,35]. Hence, numerous molecular studies indicate that the precursor of serous ovarian carcinomas may be localized in the epithelium of the fallopian tubes in the form of a precursor lesion, called serous tubal intraepithelial carcinoma [27,32,36–42]. Epidemiologic data are supporting this theory, since it has been reported that the risk of ovarian cancer decreases after tubal ligation or excision [43–46].

Two distinct histological subtypes of ovarian carcinomas, the OCCC and EnOC, have been directly associated with endometriosis through observational epidemiological studies [14,24,25,47–49]. Atypical endometriosis has long been proposed as the histological precursor lesion of OCCC and EnOC [50,51]. A direct pathogenic link of endometriosis with OCCC and EnOC has been evidenced by the important study of Wiegand *et al.* demonstrating common truncating mutations and loss of protein expression of the *ARID1A* tumor suppressor gene in OCCC and contiguous atypical endometriosis [1]. Whole-exome sequencing performed independently by Jones *et al.* in eight OCCC confirmed frequent mutations of *ARID1A*, as well as *PIK3CA*, *PP2R1A* and *KRAS*. Validation of these results in 42 OCCC (the eight tumors of the discovery cohort and an additional 34 OCCC samples as a validation cohort) by Sanger sequencing reported the frequencies of these mutations at 57%, 40%, 7.1% and 4.7%, respectively [2].

OCCC is the second most common epithelial ovarian cancer subtype, and its prevalence has been shown to differ significantly between geographic regions [52]. The highest prevalence is described in Asian countries, especially in Japan, where it accounts for 15%–25% of epithelial ovarian cancer [53,54]. The prevalence in Europe and North America is significantly lower, where it accounts for 1%–13% of ovarian epithelial tumors [55–58]. Interestingly, the prevalence of endometriosis has also been reported to be higher in Asian women in some studies [59]. OCCC are reported to occur at an earlier age than serous ovarian cancer, with a median age at diagnosis of 55 years compared to 64 years [58]. Although low-stage OCCC have a relatively good prognosis, high-stage OCCC have a poorer prognosis than stage-matched high-grade serous ovarian carcinomas and are often characterized by resistance to standard carboplatin paclitaxel chemotherapy [53,54,60–64]. Interestingly, OCCC are associated with a 2.5-fold higher incidence of clinically significant venous thromboembolism than in women with other histological types of epithelial ovarian cancer [65].

The prevalence of EnOC is estimated to be somewhat lower than in OCCC and accounts for 7%–13% of epithelial ovarian cancer [56,66,67]. There is an association of endometrioid ovarian carcinomas with uterine endometrial carcinomas in 15%–20% of cases [68–72]. Similarly to OCCC, EnOC are often diagnosed as early-stage disease in younger women (median age 47 years) in comparison with serous ovarian cancer and, often, become manifest with pelvic pain, a palpable abdominal mass, abnormal vaginal bleeding and/or newly developed or increased dysmenorrhea and dyspareunia [48,73].

Finally, seromucinous borderline tumors have also been associated with endometriosis. They are a rare subtype of mucinous borderline tumors and show a distinct non-gastrointestinal-type pattern [67]. These tumors coexist with endometriosis in 30%–70% and are likely to originate from endometriotic cysts [67,74–76].

This review focuses on common genetic alterations in endometriosis and the endometriosis-associated OCCC and EnOC, with special emphasis on *ARID1A* mutations and alterations in the PI3K/AKT pathway and potential cooperative mechanisms between these pathways.

## 2. *ARID1A* Mutations

### 2.1. Background

The *ARID1A* gene encodes the AT-rich interacting domain containing protein 1A (*ARID1A*), also known *inter alia* as BAF250a or p270, which is part of a family of 15 proteins in humans that all contain a characteristic 100-amino acid DNA-binding ARID domain that binds in a sequence non-specific manner to DNA [77]. The ARID1 subfamily is a member of seven ARID subfamilies based on degree of homology of the ARID domain and the similarity between the highly variable non-ARID domain structures [78]. *ARID1A* and *ARID1B* are the only ARID1 subfamily members and are two mutually exclusive subunits of the SWI/SNF chromatin remodeling complex [79]. It is through this complex that ARID1A probably exerts its role as a tumor suppressor [80]. *ARID1A* is located at 1p36.11 [81] and encodes a large protein of approximately 250 kD that is expressed primarily (maybe exclusively) in the nucleus [77]. Its expression is cell-cycle dependent and is higher in the G_0_/G_1_-phase compared to the S- and G_2_/M-phases [82]. *ARID1A* encodes two protein isoforms (2285 and 2086 amino acids), but there is no actual knowledge about a functional difference of the two isoforms. It has been described that ARID1A is post-translationally modified through lysine acetylation and serine/threonine phosphorylation, which may potentially regulate protein expression or protein-protein interactions [77,83]. Although its role as a part of the SWI/SNF complex is the best studied interaction of the ARID1A protein, several other protein-protein interactions, such as interactions with p53 [84], with SMAD3 [84], with hormonal receptors, such as the glucocorticoid receptor [85], and others have been described. Due to its various interactions, the understanding about the functional effects of *ARID1A* mutations remains quite poor and may vary depending on different cell types. Other components of the ATP-dependent SWI/SNF chromatin remodeling complex, such as SMARCB1, have been found to be mutated in a wide variety of cancers [77]. The frequency of mutations of the different components of the SWI/SNF complex typically differs among different cancers [86].

Mutations in the *ARID1A* gene occur in a wide variety of different cancers. They have been found to be the most frequent in OCCC, followed by EnOC, but occur ubiquitously in various cancers [86]. Frequent mutations or loss of expression of *ARID1A* have also been found in endometrial carcinomas of endometrioid (loss of expression in 29%), clear cell (loss of expression in 26%) and serous histology (loss of expression in 18%) [87,88], pancreatic (mutations in 8%–45%) [89,90] and gastric adenocarcinomas (mutations in 8%–29%) [91–93], as well as in hepatocellular (mutations in 10%–17%) [94–96] and breast carcinomas (mutations in 4%–35%) [97,98].

### 2.2. *ARID1A* Mutations in Endometriosis-Associated Ovarian Carcinomas

A high frequency of *ARID1A* mutations has been detected in endometriosis-associated ovarian carcinomas. Studies undertaken by Wiegand *et al.* [1] and by Jones *et al.* [2] have reported *ARID1A* mutations in 46%–57% of OCCC and in 30% of EnOC. In the study of Wiegand *et al.*, validated RNA-sequencing results of 19 OCCC samples (18 solid OCCC tumor samples and one OCCC cell line (TOV21G)) reported three somatic nonsense mutations, two somatic insertion/deletion mutations, one somatic missense mutation (found simultaneously in a sample containing an insertion mutation) and one gene rearrangement of *ARID1A* with the neighboring gene, *ZDHHC18*, with the fusion ends mapping to a homozygous deletion involving most of *ARID1A*. These data were verified in a mutation-validation cohort of 210 samples of ovarian carcinomas, including 101 OCCC, 33 EnOC and 76 high-grade serous ovarian carcinomas, as well as a second OCCC cell line (ES2) (*cf*. Table 1). All the 65 truncating *ARID1A* mutations that were found in 47 OCCC and 8 EnOC samples were somatic.

*ARID1A* is a large gene containing 20 exons, and the mutations found by Wiegand *et al.* were distributed evenly across the whole gene, with just a few recurrent mutations between different tumors. Most of the detected mutations were truncating (nonsense or frameshift) and correlated strongly with a loss of ARID1A protein expression in the immunohistochemistry (IHC). A total of 27 (73%) of the 37 OCCC and five (50%) of 10 EnOC samples with *ARID1A* mutations showed loss of protein expression in IHC, whereas four (11%) of 36 OCCC and two (9%) of 23 EnOC samples without *ARID1A* mutations were negative for ARID1A protein expression. Since data from exon resequencing and RNA sequencing presented excellent correlation, there was no suggestion for a relevant epigenetic silencing of *ARID1A* [1].

It is interesting that only approximately 30% of the OCCC showed homozygous mutations of *ARID1A*, whereas 73% of heterozygous mutated tumors showed a loss of protein expression without loss of heterozygosity. Similar observations in other cancer types, as well as results of *in vitro* studies, therefore, suggest a haploinsufficient tumor suppressor role for *ARID1A* [1,77].

Jones *et al.* described 32 mutations in *ARID1A* that were also distributed throughout the coding region of *ARID1A* and were all predicted to truncate the protein (nine nonsense and 23 insertion/deletion mutations). Both *ARID1A* alleles were affected through loss of heterozygosity or through biallelic mutations in 10 of the 24 tumors harboring *ARID1A* mutations [2].

The frequency of loss of ARID1A protein expression in OCCC and EnOC was verified in multiple studies [99–104] and found to be consistent with the initial observations [1,2]. Due to the high frequency of their occurrence, mutations of *ARID1A* are regarded to be one of the major genetic alterations in endometriosis-associated OCCC and EnOC [67].

An overview of the studies that analyzed ARID1A expression in ovarian cancer by mutational analysis and/or immunohistochemistry is given in Table 1.

### 2.3. Loss of ARID1A Expression in Endometriosis

Mutations of *ARID1A* have been demonstrated in atypical endometriosis that, in contrast to the adjacent OCCC tissue, was negative for HNF-1β and retained estrogen receptor expression. This indicates that *ARID1A* mutations are an early event in the pathogenesis of endometriosis-associated ovarian carcinomas. In contrast to tumor-adjacent atypical endometriosis, no mutations or loss of ARID1A expression were found in the distal non-atypical endometriotic tissue of the same patients [1].

This observation sustains the theory of *ARID1A* being a tumor suppressor in which loss of expression occurs in cell clones that are undergoing a process of precancerous alteration. However, it remains controversial at which stage of pathogenesis *ARID1A* mutations occur in endometriosis, *i.e.*, if they are limited to atypical endometriosis or if they already occur in a low-frequent manner in non-atypical endometriosis or at the early transition stage from non-atypical to atypical endometriosis.

To date, *ARID1A* sequencing studies are lacking in non-carcinoma-related endometriosis, probably due to the fact that the occurrence of *ARID1A* mutations is expected to be low in endometriosis (considering that the relative risk to developing ovarian cancer during a lifetime is approximately 1.5%) and that *ARID1A* sequencing studies are technically challenging, due to the large size of the gene and the random distribution of the mutations along the gene. Nevertheless, immunohistochemical data from different studies indicated that loss of ARID1A expression is also observable in rare cases of non-atypical endometriosis, especially in endometriotic cysts of the ovary, also referred to as endometriomas [101,104,106]. An overview of the studies that investigated ARID1A expression in endometriosis with or without relation to ovarian carcinomas is given in Table 2.

Despite the good correlation between the immunohistochemical negative ARID1A expression and its mutations (*cf*. Section 2.4.), it is not definitely clarified if these observations are the result of *ARID1A* mutations or epigenetic regulation. Sequencing analyses and further functional studies are warranted to elucidate the exact time point of the occurrence of *ARID1A* mutations and their role in (atypical) endometriosis.

### 2.4. Correlation between *ARID1A* Mutations and Loss of ARID1A Expression in Immunohistochemistry

In addition to Wiegand *et al.* [1], other groups found a strong correlation between *ARID1A* mutations and loss of ARID1A protein expression in different tumor types. Guan *et al.* [88], in addition to a large IHC analysis of 995 tumor samples of various localizations, sequenced *ARID1A* in a total of 93 tumor samples and found 10 (40%) of 25 uterine endometrioid carcinomas mutated (all of them insertion/deletion or nonsense mutations), whereas none (0%) of 12 uterine serous carcinomas and none (0%) of 56 ovarian serous and mucinous carcinomas revealed somatic mutations in *ARID1A*. They correlated the ARID1A status and IHC in the 25 uterine endometrioid carcinomas and in 51 ovarian serous carcinomas and found a significant correlation of ARID1A expression with its mutational status (*p* = 0.0014). Interestingly, they found an immunohistochemical pattern of clonal loss in *ARID1A* mutated carcinomas, which was not found in *ARID1A* wild-type carcinomas. This clonal loss of ARID1A protein expression was observed in four cases, which were classified as immunohistochemically positive, but genetically harbored *ARID1A* mutations. When combining these cases with completely negative ones, they found an even stronger correlation of mutational status and IHC (*p* < 0.0001) [88]. In a smaller study performed in OCCC, there was a concordance of ARID1A immunohistochemistry in 91% of 12 OCCC cases with known *ARID1A* mutational status with a sensitivity of 100% and a specificity of 66% [99]. In gastric adenocarcinomas, Zang *et al.* found a reduced or absent ARID1A protein expression in 75% (6/8) of *ARID1A* mutated samples and a strong positivity for ARID1A protein expression in 100% (11/11) of the samples with wild-type *ARID1A* sequence [92]. Wang *et al.* sequenced *ARID1A* in a total of 109 gastric cancers. They found 32 samples with *ARID1A* mutations, which, as discovered in OCCC and EnOC, were truncating in the majority of cases (85%). Seventy-five percent (24/32) of gastric cancers with *ARID1A* mutations immunohistochemically exhibited a loss or substantially lower ARID1A protein expression compared to cancers with the wild-type gene (*p* < 0.001) [91]. Taken together, these results suggest that an immunohistochemical loss of ARID1A expression correlates well, although not perfectly, with truncating *ARID1A* mutations, which justifies its use as a surrogate marker for the underlying gene mutations [87,101,104,107–109].

## 3. PI3K/AKT-Pathway Alterations

### 3.1. Introduction

Activation of the phosphatidylinositol 3-kinase (PI3K)/AKT pathway supports multiple mechanisms responsible for cancer progression, including proliferation, inhibition of apoptosis, cell adhesion and transformation. PI3K activation, e.g., by activating mutations of *PIK3CA* encoding the catalytic subunit, p110alpha, leads to an activation of AKT, a serine-threonine kinase, which is present in three different isoforms (*AKT1-3*) in human cancer and leads to increased cellular growth and survival of cancer cells [110]. The mammalian target of rapamycin complex 1 (mTORC1) is one of the major effectors downstream of AKT and is central in controlling cell growth and proliferation. mTORC1 was discovered through its inhibition by the drug, rapamycin [111,112]. Activation of the PI3K/AKT pathway is mainly effected by the activation of receptor tyrosine kinases and by somatic mutations in specific components of the signaling pathway. These mainly include loss of the tumor suppressor, *PTEN*, activating mutations of p110alpha (*PIK3CA*) and, less frequently, of the three isoforms of *AKT1-3*. In addition, amplifications of *AKT1* and *AKT2*, as well as of *PIK3CA* have been described in some cancers, but seem to play a subordinate role compared to the other described mechanisms [110].

### 3.2. PI3K/AKT Pathway Activation in Endometriosis

Several studies have reported PI3K/AKT pathway activation in endometriosis [113–120]. It has been shown that the PI3K/AKT pathway regulates FOXO1 protein levels, a member of the forkhead-box O family and the decidua-specific gene IGF binding protein-1 (*IGFBP-1*), which are both involved in the decidualization of endometrial cells. Levels of phospho-AKT (Ser473) were consistently higher in endometriotic stromal cells, and overactivation of PI3K/AKT led to reduced decidualization in primary endometriotic stromal cells issuing from endometriomas [113]. Reduced decidualization and IGFBP-1 secretion have also been observed in primary endometriotic cells from other localizations [121] and in eutopic endometrial stromal cells from women with endometriosis [122,123]. Decidualization of the endometrium is a remodeling event that is physiologically occurring in response to progesterone in the secretory phase of the menstrual cycle in order to prepare the endometrium for the implantation of the embryo [124]. It is known that progestins and cAMP decrease phosphorylated AKT levels and increase nuclear FOXO1 levels in eutopic endometrial stromal cells [125,126]. The response to decidual stimuli by medroxyprogesterone acetate and dibutyryl cAMP was dramatically lower in ectopic endometriotic stromal cells. Interestingly, both inhibition of PI3K and AKT led to increasing nuclear FOXO1 and IGFBP1 levels in response to treatment with medroxyprogesterone acetate and dibutyryl cAMP, supporting evidence that the increased PI3K/AKT pathway is involved in the reduced decidual response in endometriosis [113]. This observation is further interesting, since it may indicate that the PI3K/AKT pathway is involved in processes supporting the effects of progesterone resistance, a well-described characteristic of endometriosis [127]. Therefore, small molecule inhibitors may be preclinically investigated as a therapeutic option, especially in overcoming progesterone resistance in endometriosis [113].

### 3.3. PI3K/AKT Pathway Alterations in OCCC and EnOC

In contrast to high-grade serous ovarian carcinomas, where activation of the PI3K/AKT pathway through mutation of *PIK3CA*, *AKT* or inactivating mutations of *PTEN* is rather rare (< 5%), it is a clearly more frequent event in OCCC and EnOC [128]. Activating mutations in *PIK3CA* encoding p110α, the catalytic subunit of PI3K, have been described to occur in 33%–40% of OCCC [2,129]. Activation of the PI3K/AKT pathway by loss of PTEN expression has been found in 40% of OCCC [130]. Finally, *AKT2* amplification was observed in 14% of OCCC [131]. It is still unclear if aberrations in the PI3K/AKT pathway are critical drivers of cancer growth and, therefore, a possible therapeutic target in ovarian cancers [128]. Nevertheless, preclinical and clinical phase-I studies have suggested that inhibition of this pathway may help to overcome resistance to chemotherapy in ovarian cancer [132], which is a common problem in OCCC and, therefore, would be of specially great interest in this tumor type [52].

### 3.4. PIK3CA Mutations in Endometriosis-Associated Ovarian Cancer and Endometriosis

*PIK3CA* mutations, as a common mechanism of PI3K/AKT pathway activation in OCCC [133], will be discussed here in detail, since they are frequently associated with *ARID1A* mutations (*cf*. Section 4.2.).

Activating mutations in the *PIK3CA* gene, encoding the p110α subunit of PI3K, have been observed at a frequency of 33%–40% in OCCC [2,129]. Kuo *et al.* [129] first reported the high frequency of activating *PIK3CA* mutations in a large cohort of 97 OCCC, including 18 affinity-purified tumor cells from fresh specimen, 69 samples of microdissected paraffin-embedded tumors and 10 OCCC cell lines. They described an overall frequency of 33% *PIK3CA* mutations and of 46% in the 28 affinity-purified OCCCs and OCCC cell lines. The majority of the PIK3CA mutations were confined to exons 9 and 20, leading to an activation of p110α kinase. This was confirmed by immunohistochemistry, demonstrating an intense diffuse phosphorylated AKT immunoreactivity in all of the 18 specimens with *PIK3CA* mutations. This was also observed in PIK3CA wild-type tumors in 34 (85%) of 40 cases, indicating that other mechanisms are contributing to AKT phosphorylation in a large proportion of OCCCs with wild-type *PIK3CA* [129].

Yamamoto *et al.* [134] sequenced exon 9 and 20 of *PIK3CA* in 23 OCCC samples and found 10 tumors (43%) with activating mutations (H1047R in all cases). Interestingly, they observed the same mutations not only in nine adjacent atypical endometrioses of the 10 cases (90%), but they found non-atypical endometriotic tissue in six of the 10 samples (60%) with the same H1047R mutation, suggesting that these mutations occur very early in the tumorigenesis of OCCC [134].

An overview of studies that investigated *PIK3CA* mutations in OCCC and EnOC, as well as in endometriosis, is given in Tables 3 and 4, respectively.

### 3.5. Targeting the PI3K/AKT-Pathway in OCCC and EnOC

PI3K/AKT/mTOR signaling is frequently altered in EnOC and OCCC, but it is unclear whether these genetic changes are critical drivers for these carcinomas. It is therefore questionable whether these molecules are susceptible to targeted inhibition and suitable for response prediction. Several PI3K/AKT/mTOR inhibitors were explored as single agents or in combination in clinical trials of different tumor types. In ovarian and endometrial cancers, significant single-agent activity with PI3K/AKT inhibitors is rarely observed [142,143]. However, it is known that PI3K/AKT activation contributes to a reduced response to chemotherapy in ovarian cancer [144] and that modulation of the PI3K/AKT/mTOR pathway is suitable for overcoming resistance to chemotherapy [132]. Thus, Mabuchi *et al.* demonstrated increased sensitivity of cisplatin-resistant OCCC cell lines to the mTOR inhibitor everolimus, compared to the cisplatin-sensitive parental OCCC cell lines [145].

To date, reliable biomarkers for the prediction of response to therapy in ovarian cancer are not in clinical use [146]. In the past, several studies have explored the effects of PI3K/AKT/mTOR inhibitors on human cancer cells. In a study of several OCCC cells, *PIK3CA* mutations did not predict the sensitivity to PI3K/AKT/mTOR inhibitors [139]. In contrast, activated AKT was a predictive marker of drug sensitivity in ovarian cancer cells, which were treated with RAD001 (everolimus) to inhibit the mTOR pathway [147].

In a recent study, seven patients with advanced ovarian carcinomas harboring a *PI3KCA* mutation were enrolled onto clinical trials that included a PI3K/AKT/mTOR inhibitor. Prior to that, all patients had experienced treatment failure with standard therapies. Two patients (2/7, 29%) responded to this targeted therapy, which was combined with a cytotoxic drug. Other gynecologic and breast carcinomas in this study cohort also demonstrated a higher response rate than patients without *PIK3CA* mutations. Despite the small sample size, the authors conclude that *PIK3CA* mutation screening is helpful in the use of PI3K/AKT/mTOR pathway inhibition. However, single-agent use seems to not be sufficient to induce a response, because *PIK3CA* mutations often coexist with other concurrent molecular aberrations. Interestingly, both responders of the ovarian cancer subset had a simultaneous MAPK pathway mutation (one each in *KRAS* and *BRAF*) [142].

It is known that PI3K/AKT/mTOR signaling is a complex process, which interacts with the RAS/RAF/MEK/ERK pathway. In this context, it was observed that *PIK3CA* mutations may predict the response to PI3K/AKT/mTOR inhibitors, whereas concomitant mutations in the MAPK pathway (*KRAS*, *NRAS*, *BRAF*) may mediate resistance [148,149]. Therefore, clinical trials investigate several strategies, like dual targeting of PI3K/AKT/mTOR and RAF/MEK/ERK pathways, as well as the combination of multi-drugs instead of single-agents [128,150].

## 4. Further Implications

### 4.1. Functional Studies about the Loss of ARID1A Expression *In Vitro* and *In Vivo*

Cancer genome sequencing studies have created a new perspective about disordered chromatin organization as a feature of cancer, showing that mutations of epigenetic regulators are occurring frequently in a wide variety of different human cancers. Although the subject of intensive study, the exact function and role of *ARID1A* as a tumor suppressor remains far from being elucidated. *In vitro* studies have suggested different roles for *ARID1A* to exert its tumor suppressive action, which are mainly through proliferation, differentiation and apoptosis [77]. Knockdown of *ARID1A* led to increased proliferation of normal ovarian surface epithelial cells [84] and disrupted differentiation of certain cell types (e.g., osteoblasts) [151]. In Jurkat leukemia cells, Fas-mediated cell death was inhibited after *ARID1A* knockdown [152]. *ARID1A*, in contrast to the mutually exclusive *ARID1B* subunit of the SWI/SNF complex, inhibited cell cycle arrest in murine preosteoblasts [153,154]. Although these findings all constitute promising new perspectives in understanding the function of *ARID1A* as a tumor suppressor, the functional consequences of *ARID1A* mutations are probably more vast, since ARID1A regulates hundreds of different genes through the SWI/SNF chromatin remodeling complex [80]. Furthermore, it is likely that *ARID1A* mutations have divergent effects, depending on different cell and tumor types in which they are present, probably also depending on the mutational landscape in different cancer types. As a result, functional studies of *ARID1A* present a substantial scientific challenge [77].

### 4.2. Evidence for Cooperative Mechanisms between ARID1A and the PI3K/AKT Pathway

Various studies are suggesting cooperating mechanisms in relation to *ARID1A* mutations. Yamamoto *et al.* described a frequent co-occurrence between activating *PIK3CA* mutations and loss of ARID1A expression in OCCC, demonstrating that 46% of ARID1A deficient tumors were harboring *PIK3CA* mutations *versus* 17% of the ARID1A expressing tumors [102]. In endometrioid endometrial cancer, a higher frequency of PI3K/AKT-pathway alterations (PTEN loss or *PIK3CA* activating mutations) was found in tumors with a loss of ARID1A expression, and the number of tumors that showed no alteration in the PI3K/AKT-pathway was 4.6-fold higher in tumors with preserved ARID1A expression (*p* = 0.042) [107]. An association between *PIK3CA* and *ARID1A* mutations has also been reported in gastric cancer [92].

It has been suggested in many studies that loss of ARID1A is usually associated with *TP53* wild-type tumors [84,91,92,155], and one study showed evidence for a direct protein-protein interaction between ARID1A and p53 [84].

A very interesting study conducted by Liang *et al.* [156] not only identified *ARID1A* as a potential driver gene in endometrial cancer, but also demonstrated that *ARID1A* mutations frequently co-occur with mutations, leading to activation in the PI3K/AKT pathway. An important observation was that siRNA knockdown of *ARID1A* in endometrial cancer cell lines *per se* led to an increased phosphorylation of AKT, indicating a regulation of the PI3K/AKT pathway activity by ARID1A [156].

A recent study in *SMARCB1*, a potent tumor suppressor with loss of expression, especially in certain rhabdoid tumor types and which is a core subunit of the SWI/SNF complex, showed persistent activation of AKT in *SMARCB1*-deficient tumor cells, contributing to survival and proliferation. Inhibition of AKT was sufficient to inhibit development of *SMARCB1*-deficient xenograft-tumors and to inhibit proliferation of *SMARCB1*-deficient cells *in vitro* [157].

Remarkable results have been presented at the 104th Annual Meeting of the American Association for Cancer Research by Guan *et al.* [158]: The authors tested the possibility of molecular dependency of *ARID1A* and the PI3K/PTEN pathway in an *ARID1A* knockout mice and *ARID1A/PTEN* double knockout mice model. After conditional depletion of either *ARID1A* (*n* = 10) or simultaneously *ARID1A* and *PTEN* (*n* = 12), the mice with knock-out of only *ARID1A* did not develop histological alterations, whereas 40% of the mice with double knock-out of *ARID1A* and *PTEN* developed poorly differentiated ovarian tumors disseminating in the peritoneal cavity and ascites. In the other 60%, the authors found hyperplasia of the ovarian surface epithelium. This shows that ARID1A inactivation in itself is not sufficient to initiate tumor development and requires a second hit, possibly consistent with an alteration in the PI3K/PTEN/AKT pathway to lead to carcinogenic transformation [158].

These observations are exciting for at least two important reasons. Firstly, it may explain the observation of ARID1A expression in non-atypical endometriosis made by several groups [101,104,106]; loss of ARID1A expression may be an early molecular event in these cases that might increase the overall risk of developing endometriosis-associated cancer, but in itself is not sufficient to initiate cancerogenesis. Secondly, it confirms that there is a close cooperative mechanism between *ARID1A* mutations and PI3K/AKT pathway alterations that might be of importance for early tumor detection, as well as in a therapeutic context. Interdependency on PI3K/AKT activation of *ARID1A* mutated tumor clones might be a process that is targetable by small-molecule inhibitors of the PI3K/AKT/mTOR pathway.

### 4.3. Possible Clinical Implications of *ARID1A* Mutations

The question of clinical implication of *ARID1A* mutations is not yet thoroughly answered. Whilst there is fast growing knowledge about the distribution of mutations of *ARID1A* and other members of the SWI/SNF complex in various cancers, functional and clinic-pathological data remain quite sparse.

Many studies have investigated *ARID1A* mutations as prognostic markers in a multitude of different cancers, such as OCCC, as well as breast, gastric and bladder cancer [91,93,97,100,102,155,159–161], but to date, none have demonstrated a consistent prognostic significance of *ARID1A* mutational or expressional status. This may be partially due to the lack of larger prospective studies [77]. On the other hand, it has also to be answered if an early loss of ARID1A expression and/or *PIK3CA* mutation represents an increased probability for developing OCCC or EnOC in endometriosis [3,101,104,106]. Last, but not least, it will be of major interest to determine whether *ARID1A* inactivation may be therapeutically exploited by targeting downstream and potentially reversible epigenetic gene expression targets altered by remodeler mutations, such as, e.g., oncogenic proteins cyclin D1 and MYC or alterations in the Hedgehog pathway signaling [77,133,162].

An interesting synthetic-lethality therapy principle involving the SWI/SNF catalytic subunit, *BRG1/SMARCA4*, frequently deficient in non-small-cell lung carcinomas by BRM-ATPase inhibitors targeting another SWI/SNF subunit, has been proposed in a recent study [163]. This demonstrates that although the effects of SWI/SNF subunit mutations are very complex and still poorly understood, their respective downstream effectors are potentially targetable by compounds, opening a wide horizon of mechanisms that may be influenced for therapeutic purposes.

## 5. Conclusions

Although still at the beginning, the current genomic characterizations of *ARID1A* mutations and functional investigations of the loss of ARID1A expression *in vitro* and in animal studies, combined with analyses of presumed cooperating pathways as, e.g., the PI3K/AKT/mTOR pathway through direct activation and mutations of *PTEN* or *PIK3CA*, opens new perspectives for potential therapeutic approaches. This is of great interest in OCCC, since this frequently endometriosis-associated ovarian carcinoma subtype is characterized by resistance to conventional chemotherapy regimens and, therefore, has a poor prognosis in advanced stages. Mutations and consecutive loss of ARID1A expression, as well as activating mutations in the PI3K/AKT pathway (through loss of PTEN expression or *PIK3CA* activation) are highly frequent in OCCC and have to be further assessed in potential therapeutic strategies. The current intensified research activity in this field promises improved understanding and relevant progress in clinically significant aspects in the near future.

## Figures and Tables

**Table 1:** Studies that investigated AT-rich interacting domain containing protein 1A (*ARID1A*) mutations and protein expression in ovarian cancer with sequencing methods and by immunohistochemistry (IHC). OCCC, ovarian clear cell carcinomas; EnOC, endometrioid ovarian carcinomas.

Authors, year of publication	Ovarian carcinoma subtypes	Loss of ARID1A protein expression	*ARID1A* mutations by sequencing methods	Ref.
Jones *et al.*, 2010	42 OCCC	-	57% somatic *ARID1A* mutations in a total of 42 OCCC	[2]

Wiegand *et al.*, 2010	18 OCCC tumor samples and 1 OCCC cell line (whole transcriptome)—discovery cohort	Loss of ARID1A protein expression correlated strongly with the presence of *ARID1A* mutations in the mutation discovery and validation cohort.	Somatic *ARID1A* mutations (3 nonsense, 2 insertion/deletion, 1 missense and 1 gene rearrangement) in the discovery cohort	[1]
	210 ovarian carcinomas and a second OCCC cell line (*ARID1A* sequencing); mutation validation cohort		*ARID1A* mutations in 55 of 119 OCCC (46%), 10 of 33 EnOC (30%) and none of the 76 high-grade serous ovarian carcinomas	
	455 ovarian carcinomas (IHC validation cohort)	Loss of ARID1A protein expression in 55 (42%) of 132 OCCC, 39 (31%) of 125 EnOC, and 12 (6%) of 198 high-grade serous ovarian carcinomas.		

Maeda *et al.*, 2010	OCCC	Negative ARID1A expression in 88 of 149 (59%) OCCC tumor samples by IHC	Sequencing of 12 OCCC tumor samples; 9 samples with *ARID1A* mutations and 3 with wild-type expression	[99]

Guan *et al.*, 2011	serous and mucinous OC	No loss of ARID1A expression in 221 high-grade serous, 15 low-grade serous, and 36 mucinous ovarian carcinomas	No *ARID1A* mutations detected in 32 high-grade serous, 19 low-grade serous and 5 mucinous ovarian carcinomas	[88]

Katagiri *et al.*, 2011	OCCC	Loss of ARID1A expression in 9 (15%) of 60 OCCC	-	[100]

Yamamoto *et al.*, 2012	OCCC	Loss of ARID1A expression in 23 (55%) of 42 OCCC	-	[101]

Yamamoto *et al.*, 2012	90 cases of primary OCCC (including 42 previously examined)	Loss of ARID1A expression in 44% of 90 OCCC samples	-	[102]

Lowery *et al.*, 2012	212 OCCC and EnOC	Loss of ARID1A expression in 34 (41%) of 82 OCCC and 62 (48%) of 130 EnOC	-	[103]

Samartzis *et al.*, 2012	136 ovarian cancer samples as study control (23 OCCC, 28 EnOC, 63 serous ovarian carcinomas, 15 mucinous ovarian carcinomas)	Loss of ARID1A expression in 5 (22%) of 23 OCCC, 13 (46%) of 28 EnOC, 7 (11%) of 63 serous ovarian carcinomas, 4 (27%) of 15 mucinous ovarian carcinomas	-	[104]

**Table 2:** Studies investigating *ARID1A* mutations and protein expression in endometriosis.

Authors, year of publication	Endometriosis samples	Loss of ARID1A protein expression	*ARID1A* mutations by sequencing	Ref.
Wiegand *et al.*, 2010	Two cases with atypical endometriosis adjacent to *ARID1A*-deficient OCCC (adjacent and distant endometriosis was investigated from both cases)	In two patients, loss of ARID1A expression were evident in the tumor and contiguous atypical endometriosis, but not in distant endometriotic lesions	*ARID1A* mutations in the tumor and contiguous atypical endometriosis, but not in distant endometriosis	[1]
Wiegand *et al.*, 2011	10 cases of atypical endometriosis	Loss of ARID1A expression in 1 of 10 samples in the atypical areas, with retention in non-atypical endometriosis	-	[87]
Yamamoto *et al.*, 2012	59 endometriotic lesions present in 90 cases of OCCC (28 cases adjacent to tumor samples)	Complete loss of ARID1A expression in 28 endometriotic samples, of those, 17 adjacent to tumor tissue	-	[102]
Yamamoto *et al.*, 2012	22 solitary benign endometriosis samples and 28 endometriosis samples (14 non-atypical and 14 atypical) issuing from 17 patients with *ARID1A*-deficient endometriosis-associated ovarian carcinomas	All the 22 non-tumor associated endometriosis samples were ARID1A positive; 12 (86%) of the 14 tumor associated non-atypical endometrioses were ARID1A-deficient, and all of the 14 atypical endometrioses were ARID1A-deficient	-	[101]
Samartzis *et al.*, 2012	74 samples of non-atypical endometriosis: ovarian (*n* = 27), peritoneal (*n* = 19); deep-infiltrating (*n* = 28); 30 samples of normal endometrium as control	Complete lack of ARID1A expression was observed in three endometriomas (*n* = 3/20, 15%) and one deep-infiltrating endometriosis sample (*n* = 1/22, 5%); in addition, clonal expression loss was observable in cases of partially negative ARID1A expression	-	[104]
Ayhan *et al.*, 2012	15 discrete endometriotic foci remote from endometriotic cyst and ovarian carcinoma; 4 ovarian endometriomas without carcinoma and 6 cases of peritoneal endometriosis as controls	All cases retained ARID1A expression	-	[105]
Xiao *et al.*, 2012	36 cases of solitary ovarian endometriosis; normal eutopic endometrium as control	Loss of ARID1A expression in 20% of benign endometriomas; normal endometrium retained ARID1A expression	-	[106]

**Table 3:** PIK3CA mutations in OCCC and EnOC.

Authors, year of publication	Samples	*PIK3CA* mutations	Ref.
Campbell *et al.*, 2004	167 primary epithelial ovarian carcinomas, of which, 40 were samples of EnOC and OCCC and 88 were samples of serous ovarian carcinomas (all coding exons of *PIK3CA* analyzed)	*PIK3CA* mutations in 8 (20%) of 40 EnOC and OCCC compared to only 2 (2.3%) of 88 in serous ovarian carcinomas (*p* = 0.001); mutation or gene amplification of *PIK3CA* was found in a total of 45% of OCCC and EnOC	[135]
Wang *et al.*, 2005	109 advanced ovarian carcinomas, including *inter alia* 2 OCCC and 5 EnOC, as well as 90 serous and 4 mucinous ovarian carcinomas (*PIK3CA* exon 9 and 20 analyzed)	A total of 4 activating missense *PIK3CA* mutations in 109 tumors were found (in 1 of 2 OCCC, 1 mucinous and 2 serous ovarian carcinomas)	[136]
Levine *et al.*, 2005	198 unselected invasive epithelial ovarian carcinomas (exon 9 and 20 analyzed)	*PIK3CA* mutations in 24 of 198 (12%) ovarian carcinomas (not significantly different between different histological subtypes)	[137]
Willner *et al.*, 2007	12 OCCC, 26 EnOC and 51 serous ovarian carcinomas	Mutations in 3 of 12 (25%) OCCC, in 3 of 26 (12%) EnOC, but in none of 51 serous ovarian carcinomas *PIK3CA* gene amplification found in 0/22 EnOC and OCCC compared to 19/94 (20%) in SC	[138]
Kuo *et al.*, 2009	97 OCCC (18 OCCC with affinity-purified tumor cells from fresh specimen, 69 microdissected tumors from paraffin tissues, 10 tumor cell lines)	*PIK3CA* mutations in 33% of the 97 OCCC (46% of the 28 affinity-purified OCCC and OCCC cell lines)	[129]
Jones *et al.*, 2010	Whole exome sequencing in 8 OCCC samples and validation in 42 OCCC (including the 8 tumor samples of the discovery cohort) by Sanger sequencing of all exon	Mutations of *PIK3CA* in 40% of the 42 tumors (a total of 17 mutations), the majority at codons 542, 545, 546 or 1,047	[2]
Yamamoto *et al.*, 2011	23 OCCC (sequencing of *PIK3CA* exons 9 and 20)	*PIK3CA* mutations in 10 (43%) of 23 OCCC (H1047R mutations in the kinase domain in all cases)	[134]
Yamamoto *et al.*, 2012	42 OCCC (28 endometriosis-associated cases and 14 clear-cell adenofibroma-associated carcinoma cases (sequencing of exons 9 and 20)	17 (40%) of the 42 OCCC harboring *PIK3CA* mutations (majority of them ARID1A-deficient carcinomas (71%), suggesting frequent co-occurrence of mutations in these two genes	[101]
Yamamoto *et al.*, 2012	90 cases of OCCC (including 42 cases previously examined in [101]; sequencing of *PIK3CA* exons 9 and 20)	*PIK3CA* mutations found in 34 (39%) of 88 informative OCCC cases	[102]
Rahman *et al.*, 2012	Mutational analysis of *PIK3CA* (exons 1, 9, 20) and immunohistochemistry for phospho-AKT and -mTOR in 56 OCCC samples 13 ovarian carcinoma cell lines (4 serous, 9 clear cell) for *in vitro* inhibitor studies	Missense mutations in 16 (28.6%) of 56 OCCC tumor samples No correlation of *PIK3CA* mutations with the immunohistochemical pattern of phosphorylated AKT or mTOR No correlation of *PIK3CA* mutations with sensitivity to PI3K/AKT/mTOR inhibitors in OCCC cell lines	[139]
McConechy *et al.*, 2013	Select exon capture sequencing in 33 EnOC samples in addition to 307 endometrial endometrioid carcinomas	12 (40%) of 30 EnOC mutated in *PIK3CA*. 107 (39%) of 307 low-grade endometrial endometrioid carcinomas mutated in *PIK3CA*	[140]

**Table 4:** PIK3CA mutations in endometriosis.

Authors, year of publication	Samples	*PIK3CA* mutations	Ref.
Laudanski *et al.*, 2009	Gene expression study using micro fluidic gene array in eutopic endometrium of 40 women with endometriosis and 41 controls without endometriosis	*PIK3CA* expression in ovarian endometriosis significantly increased compared to endometrium of same patient. *PIK3CA* in endometrium of patients with endometriosis expressed at same level as in control endometrium. No mutations examined	[116]
Yamamoto *et al.*, 2011	Tumor-adjacent endometriotic epithelium in 10 (of totally 23 OCCC) that harbored mutations in *PIK3CA* (sequencing of *PIK3CA* exons 9 and 20)	Same H1047R mutation found in endometriotic epithelium adjacent to OCCC in 9 (90%) of 10 cases In 6 of the 9 lesions, the same mutation was found even in nonatypical endometriotic epithelium, indicating that *PIK3CA* are occurring very early in the tumorigenesis of OCCC	[134]
Vestergaard *et al.*, 2011	23 ectopic endometriotic samples (*PIK3CA* exon 9 and 20)	No *PIK3CA* mutations detected in this collective	[141]
